# Effective connectivity determines the critical dynamics of biochemical networks

**DOI:** 10.1098/rsif.2021.0659

**Published:** 2022-01-19

**Authors:** Santosh Manicka, Manuel Marques-Pita, Luis M. Rocha

**Affiliations:** ^1^ Center for Social and Biomedical Complexity, Luddy School of Informatics, Computing and Engineering, Indiana University, Bloomington, IN, USA; ^2^ Instituto Gulbenkian de Ciência, 2780-156 Oeiras, Portugal; ^3^ Universidade Lusófona, CICANT and COPELABS, Campo Grande 388, 1700-097 Lisbon, Portugal; ^4^ Binghamton University, State University of New York, Binghamton, NY, USA

**Keywords:** criticality, network dynamics, controllability, automata networks, biochemical regulation and signalling

## Abstract

Living systems comprise interacting biochemical components in very large networks. Given their high connectivity, biochemical dynamics are surprisingly not chaotic but quite robust to perturbations—a feature C.H. Waddington named canalization. Because organisms are also flexible enough to evolve, they arguably operate in a *critical* dynamical regime between order and chaos. The established theory of criticality is based on networks of interacting automata where Boolean truth values model presence/absence of biochemical molecules. The dynamical regime is predicted using network connectivity and node bias (to be on/off) as tuning parameters. Revising this to account for canalization leads to a significant improvement in dynamical regime prediction. The revision is based on *effective connectivity*, a measure of dynamical redundancy that buffers automata response to some inputs. In both random and experimentally validated systems biology networks, reducing effective connectivity makes living systems operate in stable or critical regimes even though the structure of their biochemical interaction networks predicts them to be chaotic. This suggests that dynamical redundancy may be naturally selected to maintain living systems near critical dynamics, providing both robustness and evolvability. By identifying how dynamics propagates preferably via effective pathways, our approach helps to identify precise ways to design and control network models of biochemical regulation and signalling.

## 1. Introduction

The complex organization and dynamics of living and social systems have been successfully studied with networks [1,2]. A network model of a complex multivariate system is defined by a graph G≡(X,E), where *X* is a set of nodes (variables) and *E* is a set of edges (interactions). While the structure of G reveals important properties of the organization of complex systems, we must consider dynamics to be able to predict and control their behaviour [3,4]. The simplest way to model interdependent nonlinear dynamics is with multivariate discrete dynamical systems, also known as automata networks. Boolean networks (BNs) are the simplest of such canonical models of complex systems, and exhibit a wide range of dynamical behaviours [5,6]. A formal definition is provided in §4, but let us now summarize their key features. Each node or variable in a BN is a Boolean automaton *x*_*i*_ ∈ *X*, which can take two states: *x*_*i*_(*t*) = {0, 1}, which indicate, respectively, the absence or presence of the variable at time *t* in the system dynamics;^1^ the state of *x*_*i*_ (*t*) changes (transitions) according to the state of *k*_*i*_ input nodes at *t* − 1. Logical rules specify the causal mechanisms that lead to state changes and are derived from qualitative (coarse-grained) molecular data, capturing the combinatorial regulation that is pervasive in biochemical networks [7–12]. Perhaps the key advantage of using BNs to model biomedical regulation and signalling is precisely that, unlike more traditional continuous dynamical systems, they do not require large amounts of detailed molecular data. Instead, qualitative thresholds are used to measure transitions in concentration/expression of biochemical molecules in experimental data without the need for precise parameter estimation [5,7]. Indeed, BNs have been successfully used to study the dynamics of biochemical regulation [13], cell signalling [14], metabolism [15], anticancer drug response [16] and neuronal action potentials [17], among other biomedical phenomena [7]. Dynamics in BNs ensue as the configuration of all network nodes *x*_*i*_ ∈ *X* is updated synchronously or asynchronously at time *t* until the network eventually settles into an *attractor*.^2^ An attractor can be a stable fixed-point—a configuration of node states that leads to itself in the next time step—or a sequence of configurations repeated periodically. Attractors correspond to stable biochemical states such as cell type, cell fate and healthy and disease conditions [9,10,18–20]. Famous examples include the yeast cell cycle BN that reproduces natural dynamical trajectories from known initial conditions [21], an intracellular signal transduction in a breast cancer BN that reproduces known drug resistance mechanisms and has uncovered new drug interventions [22] and a BN model used to reprogramme differentiated cells [23].

In addition to modelling specific, experimentally validated systems of biochemical regulation and signalling [24], BNs are an established modelling framework to study general properties of complex systems, including important principles of theoretical biology such as robustness and evolvability, and the two key concepts that are central to this study: *criticality* and *canalization* [6,18,25–28].

### 1.1. Criticality and the structural theory of criticality

The notion of criticality stems from physics, specifically from (and in analogy with) the observation of critical points in thermodynamic transitions between states of matter, which are controlled by some *critical* parameter (e.g. critical temperature). Tuning this parameter makes the system undergo phase transitions. When studying phase transitions in multivariate dynamical systems [18,29,30] we are typically interested in an *ordered* or stable phase, where the system dynamics is insensitive to perturbations and changes in initial conditions, and a *chaotic* phase, where dynamic trajectories are very sensitive to slight perturbations and changes in initial conditions. Thus, the transition of interest lies in a *critical* phase—between order and chaos—where the dynamics is robust to small perturbations, yet sensitive to some, making it flexible to respond differently to a range of inputs. It has been argued that complex networks (including BNs and cellular automata) need to exist in the critical phase to be able to perform collective information processing, as only in that regime do the long transients and repeating patterns necessary for long-range communication and memory exist [18,29–33] and information transfer is maximized [34,35].

This notion has similarly been used to think about the characteristics of biochemical networks that are necessary to support life. In particular, we know that evolvability requires a trade-off between phenotypic stability (for life to be robust to perturbations) and the ability to generate novelty from genetic mutations [36,37]. In other words, living systems cannot be so robust to perturbations that they cannot evolve, but cannot be so responsive to changes that they cannot persist. This has led to the idea that biochemical networks (and the living systems they support) ought to exist in a critical dynamics phase. Indeed, this idea is at the centre of Kauffman’s introduction of BNs to study the so-called *attractor hypothesis*: that stable configurations in BNs are akin to stable states in biochemical regulatory networks. From simulations, Kauffman and others further hypothesized that biochemical components in regulatory networks should have about two regulators on average, to be able to operate in a critical regime between order and chaos [18]. Recently, Bornholdt & Kauffman revisited this work and noted that the attractor hypothesis has become an accepted fact [19]. They also examined the evidence for the *criticality hypothesis*, highlighting the following findings: (i) the distribution of genes damaged by the spreading effects of deleting genes in a yeast mutant has a power-law distribution, indicating criticality [38,39]; (ii) similar initial configurations in macrophage regulatory dynamics follow parallel trajectories; these trajectories are neither identical (ordered) nor divergent (chaotic) [40]; and (iii) a large battery of 67 Boolean models of biochemical networks operate in the critical regime based on the analysis of their structure and small dynamic perturbations [41]. Indeed, it is now widely accepted that biochemical networks are critical [42–46]. See Roli *et al.* [47], and Muñoz [48] for recent reviews of the evidence for criticality in living systems.

From this backdrop, several methods have been proposed to quantify criticality and identify its critical parameters in complex multivariate dynamical systems, such as complex networks. Focusing on BNs, Derrida & Stauffer defined what we refer to as the *structural theory* (ST) of criticality for BNs [49], which defines the following surface as the critical boundary between ordered and chaotic dynamics:

1.1 2kp(1−p)=1.

It is based on two critical parameters of BNs: in-degree *k*, which is the number of inputs to each node, and bias *p*, which is the probability that an automaton node goes ON, or *P*(*x* = 1). The theory was originally defined for homogeneous BNs, where each node has the same *k* and *p*, but it has since been shown that it also holds for heterogeneous networks where in-degree and bias are randomly sampled from normal distributions with mean *k* and *p* [28].^3^

While equation (1.1) is theoretically well founded, we show below that it is not an accurate predictor of the dynamical regime, especially if the BN dynamics is in the critical regime. Before that, let us point out that we follow Derrida & Pomeau in how we measure the dynamical regime of BNs [50]. Specifically, we use the *Derrida parameter* *ζ* derived from the divergence of dynamical trajectories of the same BN after small perturbations to an initial configuration. This divergence is measured as the average number of different node-states (Hamming distance) that separate two initial trajectories (which differ in the perturbation to a single node) after *n* time steps. The *ζ* parameter is the slope of the curve of the divergence for each *n* (Derrida plot) at the origin. If *ζ* < 1, the BN is classified in the ordered regime; if *ζ* > 1, it is classified as chaotic. Thus a value *ζ* ≈ 1 indicates criticality (see §4 for details).

### 1.2. Canalized network dynamics

Waddington introduced the concept of *canalization* [51] to characterize the buffering of genetic and epigenetic perturbations that lead to the stability of phenotypic traits [52]. Recent experiments show that regulatory interactions in genetic networks are often highly canalizing in Waddington’s sense [41,44,45,53]. Automata networks have been used to formalize and study canalization theoretically and experimentally [52]. In this context, canalization is formally equated with *dynamical redundancy* in the state transition rules of automata, whereby node variables are robust to dynamic perturbations from many of their input variables, but highly responsive to just a few [6]. Such dynamical redundancy is a ubiquitous hallmark of BNs that has been used to study *canalization* in biological complexity [24,25,54]. Redundancy is linked with robustness of collective network dynamics, which contributes to stability [45,54–57], modularity [25] and controllability [4].

Canalization reveals that biochemical interactions are not equally effective in transmitting signals across regulatory networks [25]. Some interaction edges become entirely redundant, or, conversely, essential in the dynamical trajectories to attractors. This shows that the original interaction structure (graph connectivity) does not describe the real way signals propagate. Indeed, a very large ensemble of multivariate dynamical systems can fit the same interaction graph [4]. However, by taking into account the canalizing logic of automata, an underlying *effective graph* can be revealed which better characterizes the causal interactions that control cellular signalling and regulation [24]. Therefore, it is important to study exactly how canalization (dynamical redundancy) affects criticality in both random and experimentally validated biological BN models.

Previous studies of the effects of canalization on network stability and criticality have focused on *strictly canalizing* state-transition rules [6]. These are automata where one input—in at least one possible state—is sufficient to determine the state transition. Daniels *et al*. [41] have considered a linear measure of canalization, the average *sensitivity*, to study the effect of strictly canalizing functions on BN criticality. In simple terms, this measure quantifies the independent effect of each input in causing the automaton to transition, subsequently adding or averaging the contributions of each input at the node [58] and network [57] levels (see §4 for more formal definition). Notably, the average *network* sensitivity was shown to constrain the two terms of the ST defined in equation (1.1) for predicting criticality [41]. That is, criticality depends not only on the network connectivity (*k*) and automata bias (*p*), but also on the logic of the automata in network—quantified by Daniels *et al.* as the covariance between the two terms of equation (1.1): *k* and *p*(1 − *p*). Analysis of this covariance further revealed that ‘biological regulatory networks have an overabundance of canalizing Boolean functions, meaning that these functions have at least one input that can be fixed to a value that forces the output to a specific value regardless of the other inputs’ [41].

While only a few Boolean automata are strictly canalizing, most contain some amount of *collective* canalization: present when a subset of inputs, in some state combination, jointly determines an automaton’s state transition [6]. In other words, canalization is a much more frequent and nonlinear phenomenon when we consider collective canalization and not just strictly canalizing automata. Indeed, only the two parity functions for any *k* have no redundancy whatsoever in their logic (e.g. the exclusive OR, XOR, function and its negation for *k* = 2) [6,25]. Thus, to thoroughly study the effect of canalization on criticality below we introduce a new theory of criticality based on *effective connectivity*, *k*_*e*_ (*x*), as a measure of all the canalization in the logic of an automaton *x*. It is a measure of the mean number of inputs that is sufficient to determine all state transitions of *x* [25]—*k*_*e*_ is a probabilistic parameter (not a sampled statistic) of the canalizing logic of automata [24]. Importantly, effective connectivity accounts for both strict and (nonlinear) collective canalization. By contrast, as detailed in §4, sensitivity does not quantify the nonlinear or collective effects in the canalizing logic of automata (see also §3). Therefore, our new theory provides a complete characterization of the canalization phenomenon in BNs, which leads to a very significant improvement in the prediction of criticality in both random and experimentally validated biochemical regulation networks.

## 2. Results

### 2.1. The canalization theory of criticality

We approach developing a new canalization theory of criticality with a hybrid deductive and inductive (data-driven) approach. The hypothesis is that effective connectivity, as a measure of the full canalization phenomenon, captures both the connectivity and canalizing logic of automata networks better than the structure parameters used in equation (1.1). In order words, if we use *k*_*e*_ to substitute *k* and even *p* in the ST, we predict the dynamical regime of a BN more accurately. Therefore, we inductively search the space of possible ‘criticality laws’ by optimizing for the prediction (classification) of criticality using machine learning (see §4 for details). To focus on the hypothesis, we also constrain the form and complexity of the equations according the current theoretical knowledge of the problem, namely by restricting our search to the three parameters (*k*, *p*, *k*_*e*_) and the known symmetry of *p*—this constitutes the deductive component of our approach.

The new theory is searched by optimizing six model classes of increasing complexity (and parameter interaction), shown in equation (4.1) in §4. Each class is used as a binary classifier using logistic regression (with cross-validation) to best predict the dynamical regime of large *random Boolean network* (RBN) ensembles. Each class accounts for connectivity with a general parameter *κ*, allowing us to directly compare the original interaction connectivity with the effective connectivity that takes into account canalization. Specifically, for each complexity class there is a model that uses the original connectivity, *κ* ≡ *k*, and another that uses *κ* ≡ 〈*k*_*e*_〉. The bias parameter *p* is the same for both instances; see §4.

The effective connectivity of a homogeneous BN is easily computed as the mean effective connectivity of all its nodes: 〈*k*_*e*_〉 $=1/|X|.\sum_{x\in X}k_{e}(x)$. As detailed in §4, to construct RBN ensembles, nodes *x* are sampled from a catalogue of automata whose effective connectivity varies uniformly in a small interval: *k*_*e*_(*x*) ∈ 〈*k*_*e*_〉 ± *ε*, with *ε* = 0.25. RBN ensembles are further parameterized by a homogeneous bias parameter *p*—or rather by the compound term *p*(1 − *p*), given the principle of bias symmetry in logical rules. In summary, each RBN ensemble is very homogeneous and characterized by three parameters: *k*, *p* and 〈*k*_*e*_〉. For every network, *k* and *p* are constant, and effective connectivity is constrained to bins of size Δ*k*_*e*_ = 0.5 around a given 〈*k*_*e*_〉. Thus, in addition to structure (*k*, *p*), RBN are also characterized by their canalization logic (〈*k*_*e*_〉).

The dynamical regime of each BN is in turn inferred by the value of its Derrida parameter, *ζ*: if *ζ* > 1 the BN is considered to be in the chaotic regime, and ordered/critical otherwise (see §4). This classification of the dynamical regime provides (ground-truth) labels to measure the dynamical regime classification performance of each (logistic regression) model and model class—the possible ‘criticality laws’. Out of the 266 400 RBNs in our ensembles, 224 083 (approx. 84%) are classified as chaotic. Therefore, cross-validation prediction performance is best captured by measures tailored for unbalanced classification scenarios such as the *Matthews correlation coefficient* (MCC) [59]. We also show results for McFadden’s *R*^2^ since we are performing logistic regression and the *area under the curve* (AUC) for ranking performance; see §4 for details.

**Figure 1.**
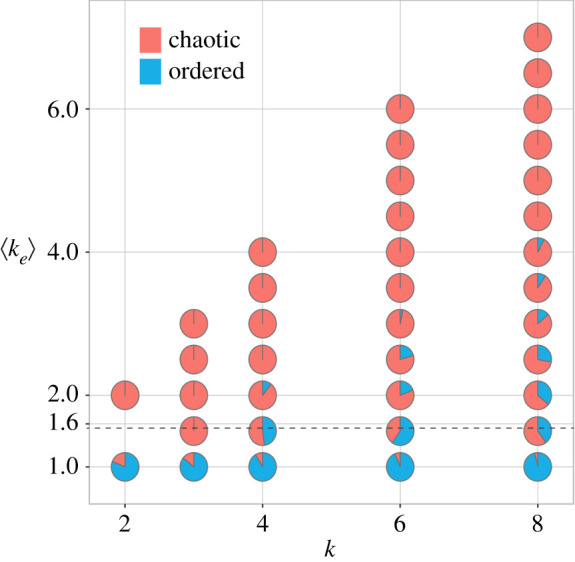
Dynamical regime of RBN in the (*k*, 〈*k*_*e*_〉) parameter space. Pie charts depict the dynamical regimes of RBN ensembles for each possible (*k*, 〈*k*_*e*_〉) pair. Blue and red areas indicate the proportions of networks with ordered and chaotic dynamics, respectively. The black dashed line corresponds to the critical effective connectivity: 〈*k*_*e*_〉^*c*^ = 1.59. Note that any decision surface with interaction between *k* and 〈*k*_*e*_〉 (any curve in a plane distinct from the horizontal 〈*k*_*e*_〉^*c*^ = 1.59) would not improve separation of the dynamical regime as pie charts above 〈*k*_*e*_〉^*c*^ = 1.59 have a majority of chaotic networks. This explains why a regression with the interaction term does not improve classification performance (see main text).

**Figure 2.**
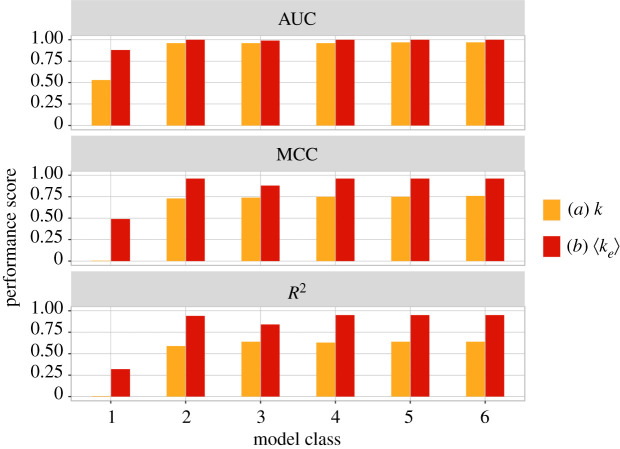
Performance measures for the regression models used to find the optimal critical boundary. Each model belongs to one of six model classes—labelled in increasing order of complexity (equation (4.1)). For each model class, orange refers to the optimal model based on *k* as a tuning parameter, and red refers to the optimal model based on 〈*k*_*e*_〉. For every model class and performance measure, 〈*k*_*e*_〉 outperforms *k* significantly (see also figure 3).

The lowest complexity model class (1) is used to compare the predictive power of *k* and 〈*k*_*e*_〉, disregarding bias *p*. It yields the following optimal decision boundaries: −*βk* = 1 and 0.63〈*k*_*e*_〉 = 1. The corresponding critical values for the tuning parameters are *k*^*c*^ = 0 and 〈*k*_*e*_〉^*c*^ = 1.59. The model instance based on *k* classifies every BN as chaotic,^4^ whereas the instance based on 〈*k*_*e*_〉 partitions the data into two reasonably correct dynamical regimes. Effective connectivity (〈*k*_*e*_〉) is a much better predictor than in-degree (*k*) for this model class, as can be seen in figure 1. The (cross-validation) classification performance is shown in figure 2, with model class (1) depicted in the leftmost column. It is clear that the model based on the original interaction structure cannot discriminate the dynamical regime of BNs at all, with MCC(*κ* ≡ *k*) ≈ 0. By contrast, the model based on effective structure alone leads to a reasonable classification performance MCC(*κ* ≡ 〈*k*_*e*_〉) ≈ 0.49, even without using *p*; similar behaviour is observed for *R*^2^. Moreover, AUC(*κ* ≡ *k*) ≈ 0.5, while AUC(*κ* ≡ 〈*k*_*e*_〉) ≈ 0.88. Thus, the best classifier based solely on in-degree *k* is equivalent to a random coin toss, while the best classifier based solely on effective connectivity *k*_*e*_ yields reasonably good performance.

To test whether *k* and *k*_*e*_ synergize to predict criticality, we performed a logistic linear regression with an interaction term between *k* and 〈*k*_*e*_〉, which model class (1) does not consider. The optimal critical decision boundary obtained is 0.1*k* + 0.7〈*k*_*e*_〉 − 0.1*k*〈*k*_*e*_〉 = 1. However, there is no improvement in MCC ≈ 0.49, which demonstrates that *k* does not provide additional information about BN criticality that is not already included in 〈*k*_*e*_〉. The coefficient of 〈*k*_*e*_〉 in the optimal model is seven times larger than that of *k*, which further highlights the relevance of each parameter in predicting BN criticality. In summary, the optimal models from class (1), and the lack of synergy between *k* and *k*_*e*_, demonstrate that the original network connectivity on its own carries no information about criticality exceeding that of *k*_*e*_—and that *k*_*e*_ incorporates almost all the necessary connectivity information. This result strongly suggests that canalization plays an important role in criticality.

Model class (2) is defined in equation (4.1) by the interaction between the bias parameter *p*(1 − *p*) and either *κ* ≡ *k* or *κ* ≡ 〈*k*_*e*_〉. The optimal decision boundaries obtained for each instance are, respectively,

2.1 $$c_{1}kp(1-p)=1,\;c_{1}=1.49$$

and

2.2 $$c_{1}\left\langle k_{e}\right\rangle p(1-p)=1,\;c_{1}=3.94.$$

**Figure 3.**
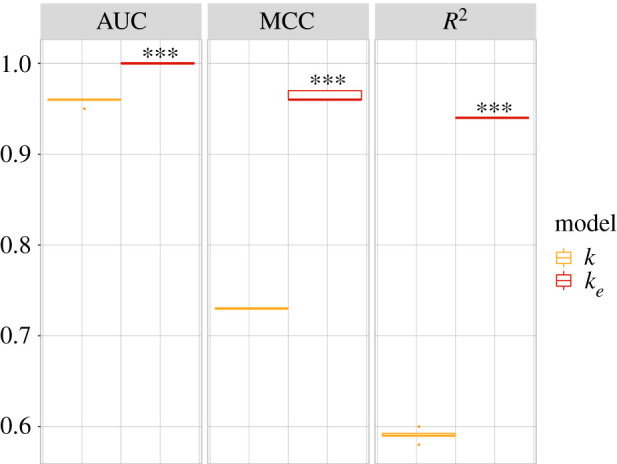
Classification performance of the two model instances in class (2) under nested fourfold cross-validation. Significant differences (*P* < 0.001) are indicated with ‘***’, after a one-sided paired-sample *t*-test.

The corresponding performance metrics are shown in the second column of figure 2. It is clear that the model class (2) instance with *κ* ≡ 〈*k*_*e*_〉 outperforms the instance with *κ* ≡ 〈*k*_*e*_〉 substantially. Indeed, the decision boundary of equation (2.2) leads to near-perfect MCC = 0.96 and *R*^2^ = 0.94 scores, and perfect ranking performance measured by AUC ≈ 1. By contrast, the decision boundary of equation (2.1), which represents the optimal, empirically derived ST,^5^ leads to significantly lower classification performance for all three performance measures, as shown in figure 3.

**Figure 4.**
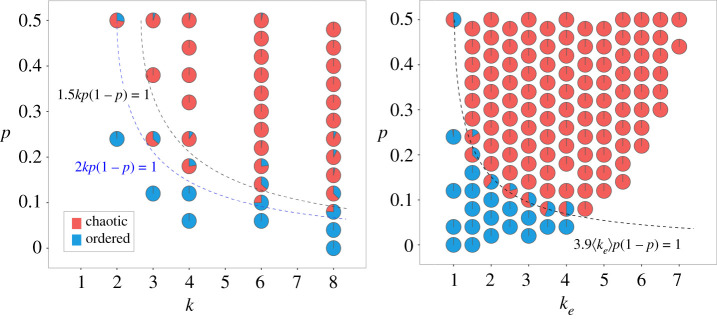
Dynamical regimes of RBN ensembles in the (*k*, *p*) and (〈*k*_*e*_〉, *p*) parameter spaces. Pie charts depict the dynamical regimes of RBN ensembles for each possible (*p*, *k*) or (*p*, 〈*k*_*e*_〉) pair, on the left and right panels, respectively. Blue and red areas indicate the proportions of networks with ordered and chaotic dynamics, respectively. Black dashed curves show the optimal criticality decision surfaces for model class (2): equation (2.1) on the (*k*, *p*) space is shown on the left panel, and equation (2.2) on the (〈*k*_*e*_〉, *p*) space is shown on the right panel. The dashed blue curve shown in the left panel corresponds to the theoretically derived ST given by equation (1.1).

The difference between the two instances is clearly observed in figure 4. Indeed, there are striking differences in how the RBN ensembles are projected onto the phase transition spaces associated with each model instance. In the effective connectivity space (right), a very crisp boundary exists between stable and chaotic dynamics which is optimized by equation (2.2); the two regimes are neatly separated with almost no misclassifications on either side of the critical boundary. This is in sharp contrast to the more uncertain boundary observed in the original connectivity space (left). This is true for both the theoretically derived ST (equation (1.1)) and the experimentally optimized version (equation (2.1)). In particular, note that stable networks are observed well into the predicted chaotic regime, and vice versa.

The classification performance (figures 2 and 3) together with the observation of the arrangement of dynamical regimes around the critical boundaries in figure 4 demonstrate that using effective connectivity instead of the original connectivity of RBNs leads to a much more accurate, near-perfect prediction of the critical boundary that separates stable and chaotic dynamics, as well as a more organized characterization of both regimes. In other words, considering the effect of canalization and interaction bias at the micro-level leads to an optimal prediction of macro-level dynamics. Indeed, model 2 with *κ* ≡ 〈*k*_*e*_〉 (equation (2.2)) yields the optimal critical boundary, as discussed below. Therefore, henceforth we refer to this model, given by equation (2.2), as the *canalization theory* (CT) of criticality in BNs.

### 2.2. The CT optimizes model complexity and classification performance

**Figure 5.**
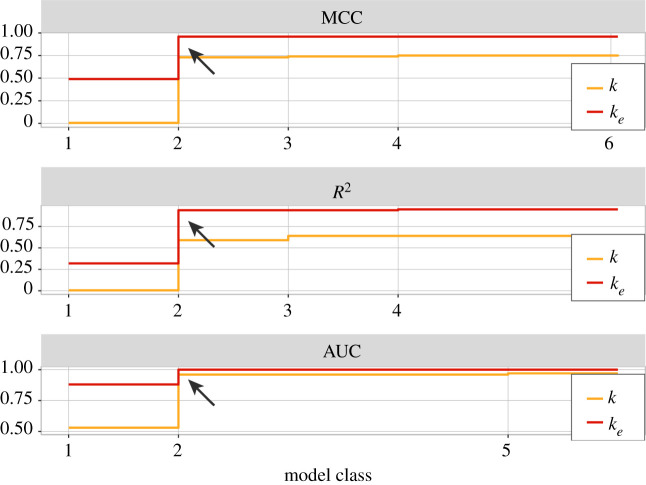
Pareto front analysis of model complexity versus classification performance for the six model classes fitted to RBN ensembles. Models are in increasing order of complexity from class 1 to class 6 as per equation (4.1) (§4). A model class is labelled on the horizontal axis only if its performance is greater than the performances of all models of lower complexity for either model instance. For each model class, orange illustrates *κ* ≡ *k* as a tuning parameter, and red *κ* ≡ 〈*k*_*e*_〉 instead. Arrows mark the performance of the optimal model class, characterized by a substantial rise followed by marginal or no gain afterwards. Note that, for all performance measures, model class 2 with 〈*k*_*e*_〉, our CT, yields the best Pareto front performance.

We use a Pareto front analysis to identify the models from equation (4.1) that best balance the trade-off between model complexity and dynamical regime classification performance. This method relies on the graphical representation shown in figure 5, which depicts the classification performance (vertical axis) against the different model classes ordered by increasing complexity (horizontal axis). Model 2 with *κ* ≡ 〈*k*_*e*_〉, the CT, achieves near-perfect classification performance with MCC = 0.96 and *R*^2^ = 0.94, and perfect ranking AUC ≈ 1. More complex models cannot improve much at all over such performance, leading to very marginal or no increase in classification performance. Therefore, the CT is the Pareto-optimal model for all performance measures (identified by arrows in the figure). Regarding models with *κ* ≡ *k*, even though there is much room to improve classification performance, increasing the complexity of the models does not lead to relevant performance gains. This implies that unless canalization is factored in, as in the model instances based on *κ* ≡ 〈*k*_*e*_〉, no increase in performance is gained over the (theoretical or empirical) ST. We thus conclude that model class (2) is optimal in terms of simplicity and performance for both instances, but the instance that uses effective connectivity, the CT, is significantly better at predicting the dynamical regime of BNs (see also figures 2 and 3). It should be noted that a search for ‘criticality laws’ that is not constrained to the model classes of equation (4.1) also does not identify any decision surface that outperforms the CT (equation (2.2)). We report the results of using symbolic regression [60] (see §4) in the electronic supplementary material. We used this method alongside the main method used here to discover optimal critical regime decision surfaces. Symbolic regression does not make any assumptions about the form of the decision surface. It independently identified an expression that is almost identical to the CT, which is further evidence for considering it an optimal criticality theory for predicting dynamical regime in BNs.

### 2.3. The CT generalizes to out-of-sample networks and significantly outperforms the ST

To estimate the statistical significance of the superior performance of the CT (equation (2.2)) with regard to the ST (equation (1.1) and (2.1)), as well as to ensure that it does not derive from over-fitting the RBN ensemble data, we compare both instances of models in every class (equation (4.1)) under nested fourfold cross-validation (details in §4). In other words, the reported performance measures (MCC, AUC *R*^2^) in figure 2 refer to classification performance on (repeated) out-of-sample RBNs, i.e. prediction. This demonstrates that the performance of the CT generalizes to out-of-sample data. Furthermore, all (out-of-sample) performance measures for the CT are significantly better than for the empirically derived ST (equation (2.1)), based on paired-sample *t*-tests (*P* < 0.001), shown in figure 3.^6^ Additional Vuong and Clarke tests confirm the results; see electronic supplementary material, §1 for details.

This analysis supports the assertion that the CT predicts criticality in BNs significantly better than does the ST.

### 2.4. The CT characterizes the dynamical regime of models in systems biology

To study how the CT characterizes the dynamical regime in experimentally validated systems biology models of biochemical regulation and signalling, we analyse 63 networks from the Cell Collective (https://www.cellcollective.org/) repository of such models [20]. Before studying the dynamical regime of these BN models, it is worth measuring the amount of canalization (dynamical redundancy) they contain, and how it changes their original interaction connectivity.

**Figure 6.**
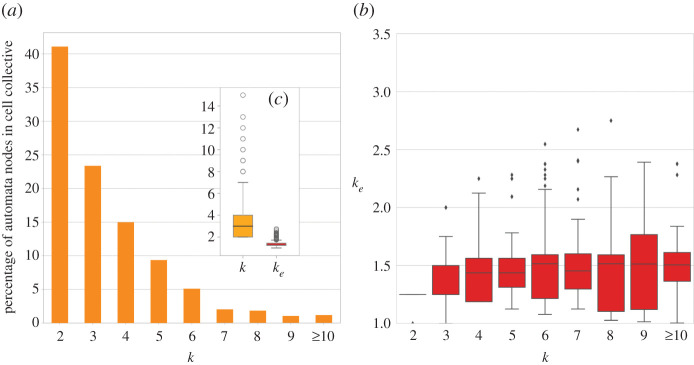
Distribution of *k* and *k*_*e*_ for automata in 63 Cell Collective BN models. (*a*) Histogram of *k*^*C*^. (*b*) Original connectivity, *k*, versus effective connectivity, *k*_*e*_, in set *C*; distributions of *k*_*e*_ per *k* shown as box plots. Automata with *k* ≥ 10 are aggregated in a single bin. (*c;* in-set) distribution of *k* and *k*_*e*_ for the entire set of cell collective automata.

The 63 BN models from the Cell Collective that we analysed comprise 2979 automata in total (after removing Boolean functions that are tautologies or contradictions). Additionally, we also removed the 48% of these automata that have a single input from the comparison since *k*_*e*_ = *k* = 1. Therefore, only 52% of Cell Collective automata have *k* > 1 and can be canalizing functions of some kind: 50% with 2 ≤ *k* ≤ 9, plus 2% with 10 ≤ *k* ≤ 15. This set, denoted by *C*, contains |*C*| = 1528 automata. Its in-degree distribution, *k*^*C*^, is shown in figure 6*a*. It is right-skewed with skewness (Pearson’s moment coefficient) ≈2, and leptokurtic, with normalized kurtosis ≥ 5. The mean, median and interquartile range are $\langle k^{C}\rangle =3.41,\tilde{k^{C}}=3$ and IQR(*k*^*C*^) = 4 − 2, respectively.

The effective connectivity distribution $\left(k_{e}^{C}\right)$ for this automata set is also right-skewed and leptokurtic, with similar skewness ≈2 and normalized kurtosis ≥ 6. However, in contrast to what is observed for *k*, the central tendency of effective connectivity is considerably smaller and its dispersion much narrower: $\langle k_{e}^{C}\rangle =1.34$, $\tilde{k_{e}^{C}}=1.25$ and $\mathrm{IQR}(k_{e}^{C})=1.43-1.25$.^7^ In other words, while the original interaction structure of these network models would lead us to infer that the mean (median) number of regulators of a biochemical variable is 3.4 (3), in reality, accounting for the canalizing dynamics reveals that the mean (median) number of *effective* regulators is only 1.34 (1.25)—i.e. on average, only 1.34 inputs are sufficient to control an automaton (or half of all automata do not need more than 1.25 inputs to be controlled). Moreover, while heterogeneous with high skewness and kurtosis, the dispersion of effective connectivity is circumscribed to a much smaller range than the dispersion of *k*—even the median of the upper quartile is only 1.43.^8^ In summary, effective connectivity varies heterogeneously but is much smaller and contained than the original interaction connectivity of these experimentally validated models from the Cell Collective. Because the true dynamical connectivity, as revealed by *k*_*e*_, is much smaller in these networks than their interaction structure implies, their dynamics should in turn be more stable than expected. Therefore, the CT should characterize critical dynamics in these models better than the ST, as we investigate next.

Similarly to the RBN ensembles, the dynamical regime of the Cell Collective models can be inferred from their Derrida parameter, *ζ* (§4), which varies very little: IQR (*ζ*) = 0.976 − 0.9 and range *ζ* ∈ [0.65, 1.15] (see figure S4-1 in electronic supplementary material). Only 11 (out of 63) models are in the chaotic regime *ζ* > 1, albeit very near the critical boundary since *ζ* ≥ 1.15. The other 52 models have *ζ* values slightly below *ζ* = 1, and are thus stable but also near the critical boundary. In summary, all Cell Collective models are in, or very close to, the critical regime, coherently with what is known about them [41].

**Figure 7.**
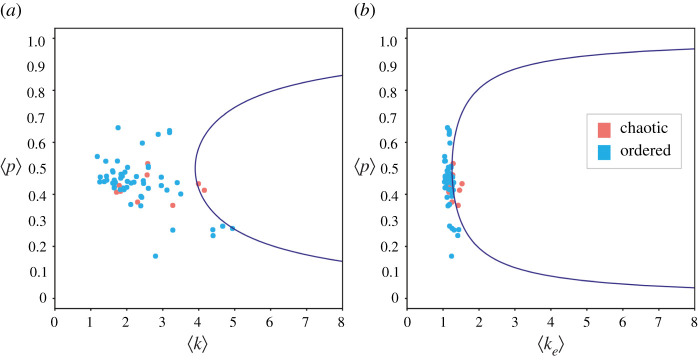
Dynamical regime of Cell Collective networks projected onto the ST (*a*) and CT (*b*) spaces. Blue dots denote stable models (*ζ* < 1), and red dots denote chaotic models (*ζ* > 1). The axes are labelled with the mean value of the relevant tuning parameters for each of the 63 BN models considered. Optimal critical boundary curves are shown in blue.

As shown in figure 7, projecting all models onto the ST space of (〈*k*〉, 〈*p*〉) does not reveal a similar dynamical regime near the critical dynamics, with networks dispersed over a large portion of the space. By contrast, the CT space (〈*k*_*e*_〉, 〈*p*〉) correctly reveals that all networks are very near each other (especially in their effective connectivity) and near an optimal critical boundary. To quantify how well each space characterizes the dynamical regime, optimal critical boundary curves are recomputed by fitting class-2 models (equations (4.1)) representing the ST and the CT to maximize the MCC score for Cell Collective models, instead of RBN ensembles as above (equations (2.1) and (2.2) and figure 4). The values of AUC obtained demonstrate that the CT space is much better correlated with the dynamical regime: AUC (ST) = 0.54 and AUC (CT) = 0.81. This shows that the ST is only marginally better than a random toss according to the AUC ranking measure, while ranking is far superior for the CT. In other words, chaotic networks are ranked above a stable network 81% of the time for the CT, but only 54% of the time for the ST. The classification performance itself is also superior for the CT, even though the many more stable than chaotic models (all very near-critical) make the classification scenario very unbalanced with the exact performance value less relevant: MCC (ST) = 0.44, MCC (CT) = 0.58.

It should be noted that the analysis of the Cell Collective models is based on assumptions made for RBNs. The CT (equation (2.2)) was developed for homogeneous networks with fixed *k* and *p*, but the Cell Collective networks are heterogeneous. Therefore, we use the mean values of these quantities in our analysis, as shown in figure 7*a*. While the CT can be properly developed for heterogeneous networks in the future (see §3), for the Cell Collective analysis we simply derived new critical boundary curves by re-fitting model class 2 (equations 4.1) using the mean value of *k*, *p* and *k*_*e*_ for each network. Interestingly, the *c* coefficients of the models optimized for the Cell Collective are not very different from those optimized for the homogeneous RBNs (equations (2.1) and (2.2), figure 4). In the (〈*k*〉, 〈*p*〉) space of the ST, *c* = 1.03 for Cell Collective networks and *c* = 1.49 for homogeneous RBNs (equation (2.1)). In the (〈*k*_*e*_〉, 〈*p*〉) space of the (*CT*) =, *c* = 3.2 for Cell Collective networks and *c* = 3.93 for the homogeneous RBNs. The change in *c* results in a shift of the critical boundary slightly to the right in the case of the heterogeneous networks of the Cell Collective, thus increasing the area of the stable regime. This is an expected result, since we know that heterogeneous connectivity leads to more stable BN dynamics [28].

Overall, and in summary, it is clear that including information about canalizing dynamics in a model of criticality yields a substantially better correlation with (cf. AUC score) and prediction of (cf. MCC score) the dynamical regime of both RBNs and systems biology automata network models.

## 3. Discussion

### 3.1. The CT based on effective connectivity is more accurate in predicting criticality than the ST and belongs to the same model class

Previous studies of criticality in automata networks have relied on the ST, which characterizes networks and their critical boundary in the (*k*, *p*) space.

The CT introduced here includes the effects of node-level canalization and characterizes networks and their critical boundary in the (〈*k*_*e*_, *p*) space instead. This effective connectivity space is much more correlated with the dynamical regime than the original connectivity space for both random ensembles (figure 4) and systems biology models (figure 7). In this new space, the criticality boundary also leads to much more accurate predictions (figures 2, 3 and 5).

Notably, the CT belongs to the same model class as the ST.^9^ The Pareto-optimal model class is of the form *cκp* (1 − *p*), where the network connectivity term *κ* is the original in-degree (*k*) in the ST or the effective connectivity (*k*_*e*_) in our CT (§3). The bias of state transition rules in the network is denoted *p*, and coefficient *c* defines where the curve is positioned in the relevant parameter space (*κ* is smallest when *p* = 1/2). Thus, in both theories, the tuning of criticality depends on interaction between the connectivity and bias parameters. However, our work reveals that a correct measure of connectivity must include the canalization that influences node dynamics—how many signals truly influence each node. The effective connectivity used in the CT reveals the true (canalized) connectivity of automata networks [24] and thus, ultimately, their macro-level dynamical regime. This is demonstrated by the superior prediction performance of the CT compared with that of the ST. In other words, criticality depends not only on structural connectivity and bias but also very significantly on canalizing dynamics. Indeed, a prediction of criticality without bias (model class 1 in §2) shows that effective connectivity alone yields a reasonable prediction performance, but in-degree alone does not (§2 and figures 1, 2 and 5).

### 3.2. Effective connectivity captures characteristic properties of the dynamical regime

The ST implicitly assumes that all functions of the same *k* and *p* contribute in the same way to the dynamical regime. We demonstrate, however, that a finer characterization of the canalized logic of individual automata is necessary to accurately predict the dynamical regime of automata networks. In figure 4*a*, homogeneous networks of the same size whose nodes are automata with the exact same *k* and *p* are shown to have opposite dynamical regimes, even far from the critical boundary of the ST.^10^ By contrast, when we transform the critical phase transition space to the finer characterization enabled by *k*_*e*_, as in figure 4*b*, networks with the same *p* and 〈*k*_*e*_〉 almost always display the same dynamical regime—except very near the CT critical boundary—as demonstrated by a near-perfect MCC score (§2). Note further that in the latter case networks are not homogeneous in *k*_*e*_ and are therefore grouped by 〈*k*_*e*_〉, so some variation in dynamical regime for the same *p* and 〈*k*_*e*_〉 is expected. Even so, such variation is only observed near the critical boundary, which demonstrates that *k*_*e*_ (and its mean value 〈*k*_*e*_〉 in the BN) is very characteristic of the dynamical regime. Finally, note that *k*_*e*_ includes the contribution of collective canalization, while other measures of canalization such as *sensitivity* do not (§3). This means that the nonlinear effects of collective canalization are included [24] and contribute to the finer characterization of criticality that the CT, based on effective connectivity, provides.

### 3.3. Effective connectivity is smaller and more contained than original connectivity

This study reveals that canalized dynamics typically alters the original interaction structure of a BN. The resulting effective structure is characterized by a smaller and more contained connectivity—smaller central tendency, dispersion and range (§2). Indeed, a consistent observation in our results is that 〈*k*_*e*_〉 ≪ *k* for most automata both in the RBN ensembles and in the 63 heterogeneous Boolean models of biochemical regulation and signalling that we analysed; see figure 6*b* and S3-1 and S4-2 in the electronic supplementary material, which are also coherent with the edge effectiveness analysis in [24].

Figure 6 highlights how much smaller and contained is effective connectivity in comparison with the original interaction connectivity. Note, for instance, that even automata with *k* ≥ 10 have $\tilde{k_{e}}=1.5$ and no automaton in the data reaches *k*_*e*_ = 3. In other words, even though the interaction network leads us to infer that some biochemical variables are regulated by more than 10 other variables, in reality, once dynamical redundancy is factored in, they never need more than three regulators to be expressed or inhibited; the vast majority of automata never need more than two regulators (see electronic supplementary material, §4 for additional details). Furthermore, *k*_*e*_ is significantly smaller in Cell Collective automata than for same sized and biased random automata [24]. While some models in the Cell Collective match experimental data only partially, we assume that the dynamical behaviour of these models for initial conditions that have not been experimentally validated is similar. Since the logic of transition functions was selected such that the overall network dynamics corresponds to what is experimentally observed for validated initial conditions, the observed redundancy (node-level effective connectivity) corresponds to experimentally validated regulatory and signalling pathways anyway. The observed ubiquitous redundancy suggests that biochemical regulation and signalling dynamics is much more canalized (redundant) than experimental interaction data imply (from which Cell Collective models were derived). Indeed, dynamical redundancy as conceptualized here may be a mechanism that allows biological organisms to operate in near criticality for greater robustness and evolvability (§1).

We are aware that the ST has been extended to consider heterogeneous BNs—with, for example, power-law distributions [28,61]. We do not consider such an extension for the CT in the present work because the networks in the random ensembles and Cell Collective are not large enough to properly distinguish heterogeneous degree distributions [62]. Nonetheless, the small and contained distribution of effective connectivity we have observed occurs in both the homogeneous random ensembles and the more heterogeneous biochemical regulation and signalling networks (§2).

This suggests that larger, very heterogeneous biological regulation and signalling networks (lognormal or asymptotic power-law degree distributions) may effectively function dynamically with more contained and low-degree distributions—even if the distribution of effective connectivity remains heterogeneous within a small range (§2). An exhaustive study of the topology of effective structure is still needed to investigate this hypothesis, which we intend to do in future work with larger, more heterogeneous networks.

It is known that the effective structure of automata networks impacts the dynamics and controllability of BNs [4,25]. While effective structure can be easily computed [63] and used to uncover specific control pathways in biochemical regulation and signalling (including in response to input nodes) [24,25], we do not yet know how its topology is organized to facilitate or hinder dynamical control, including synchronization [64]. The present research demonstrates that the canalizing dynamics that defines an underlying effective structure is an important factor in determining critical dynamics in random and experimentally validated biochemical networks, suggesting that this happens because effective connectivity is much smaller and contained than the original interaction connectivity (§2).

### 3.4. Beyond criticality: harnessing canalization in complex systems

The theoretical developments and experimental results we present provide a new theory of criticality that accounts for canalization. Based on the same class of mathematical functions, the new theory does not increase the complexity of the current theory, but increases substantially and significantly the ability to accurately predict the dynamical regime of automata networks. Given that automata networks are canonical examples of complex multivariate dynamical systems, the high classification accuracy of the new theory strongly suggests that canalization (as dynamical redundancy) is a prime mechanism for tuning the dynamical regime of complex systems. This observation is consistent with Waddington’s notion of canalization [51], whereby most random dynamical perturbations are not effective and only a few interactions control changes in network dynamics. It suggests that evolution in biological regulation has selected for *redundancy*, which has long been hypothesized as a requirement for the trade-off between robustness to random perturbations and selective responsiveness that is necessary for evolvability [36,37]. Indeed, our results with experimentally validated systems biology models suggest that canalization plays a fundamental role in the dynamics of biochemical regulation and signalling, which is missed by studying the structure of biochemical interactions alone. Therefore, beyond the study of criticality, a precise characterization of canalization is likely to enable the tailoring of interventions in complex systems towards desirable dynamical behaviour [4,24,25], including contributing to a better understanding of when criticality is desirable or not.

It is important to note that the work presented focuses on discrete dynamical systems cast as BNs. As discussed in §1, these systems have proven very useful to accurately model biochemical network dynamics via qualitative threshold estimation. Indeed, many biochemical regulation and signalling networks are likely to function more akin to discrete than continuous processes because information transmission via threshold dynamics is easier to implement by collections of decentralized molecules and cells than signals that depend on precise, meaningful gradations. For instance, we know that gene expression patterns do not typically have meaningful gradations of expression [65], which is why discrete, qualitative modelling of gene regulation is feasible [7]. Indeed, discrete-like, threshold dynamics are quite common in biology from neuronal firing dynamics [66], to T-cell activation [67,68] and even bacterial quorum sensing [69]. However, whether because of preference or best fit to experimental data, many biological systems may be best modelled by networks with continuous dynamics, for which there is not yet a comparable theory of canalization. We could use the Lyapunov exponent as a transition parameter between order and chaos in continuous systems, since it is related to the Derrida parameter [70]. But a measure of effective connectivity in continuous dynamical systems, indeed a theory of redundancy, remains a goal for future development. Until there is such a measure and theory, we do not know if our results will generalize for continuous multivariate dynamical systems.

The concept of effective connectivity underlying the CT integrates information about the structure and dynamics of multivariate interactions—in-degree connectivity and input redundancy in state transitions, respectively. It implies that the behaviour and function of complex systems is dictated by an *effective structure* that is revealed only after removal of causal redundancy in the logic of how biochemical variables integrate input signals. This truer structure of interactions provides a more accurate portrait of causal multivariate dynamics, which is more canalized than the original structure of interactions implies. This is why we find stable (or critical) dynamics in networks whose structure would be predicted by the current ST to be chaotic, and vice versa (figures 4 and 7). In this sense, canalization is a network-level mechanism that can be tailored by evolution to facilitate or hinder effective cross-talk in biochemical regulation and signalling [24]. Going forward, the methodology can provide powerful analytical tools to uncover the causal pathways that determine control and resilience to interventions in various complex systems [63], such as genetic regulation in biological development [25] and treatment strategies in cancer and other diseases [24].

## 4. Methods

### 4.1. Boolean automata definitions and notation

A *Boolean automaton* is a binary variable, *x* ∈ {0, 1}, where state 0 is interpreted as *false* (*off* or *unexpressed*), and state 1 as *true* (*on* or *expressed*). The states of *x* are updated in discrete time steps, *t*, according to a *Boolean state transition rule* of *k* inputs: $x^{t+1}=f\;(i_{1}^{t},\ldots ,i_{k}^{t})$. Therefore, *f* : {0, 1}^*k*^ → {0, 1}. Such a rule can be defined by a *Boolean logic formula* or by a *look-up (truth) table* (LUT) with 2^*k*^ entries. Each LUT entry of an automaton *x*, *f*_*α*_, is defined by (i) a specific *condition*, which is a conjunction of *k* inputs represented as a unique *k*-tuple of input-variable (Boolean) states, and (ii) the automaton’s *next state* (transition) *x*^*t*+1^, given the condition. We denote the entire state transition rule of an automaton *x* in its LUT representation as $F\equiv \{f_{\alpha }\;:\;\alpha =1,\ldots ,2^{k}\}$.

### 4.2. Boolean networks

A BN is a graph B≡(X,E), where *X* is a set of *n* Boolean automata *nodes* *x*_*i*_ ∈ *X*, *i* = 1, …, *n*, and *E* is a set of directed edges *e*_*ji*_ ∈ *E* : *x*_*i*_, *x*_*j*_ ∈ *X*. If *e*_*ji*_ ∈ *E*, then automaton *x*_*j*_ is an input to automaton *x*_*i*_, as computed by *F*_*i*_. *X*_*i*_ = {*x*_*j*_ ∈ *X* : *e*_*ji*_ ∈ *E*}, which denotes the set of input automata of *x*_*i*_. Its cardinality, *k*_*i*_ = |*X*_*i*_|, is the *in-degree* of node *x*_*i*_, which determines the size of its LUT, $|F_{i}|=2^{k{\;}_{i}}$. We refer to each entry of *F*_*i*_ as *f*_*i* : *α*_, $\alpha =1\cdots 2^{k{\;}_{i}}$. Not all nodes in a BN are regulated by other nodes in the same network. Some nodes can be *input nodes* that act as regulators of other nodes, but that are not regulated by nodes in the network. In biochemical network models such nodes are used to capture the regulatory effects of external factors such as temperature, biochemical signals and others, which have state transitions regulated outside the network. Input nodes are often modelled as nodes that remain fixed in their initial state throughout simulations of BN dynamic trajectories, i.e. they are modelled as nodes that regulate themselves through a simple self-loop, and thus *k* = 1. At any given time *t*, B is in a specific *configuration* of node states, ***x***^*t*^ = [*x*_1_, *x*_2_, …, *x*_*n*_]. We use the terms *state* for individual automata (*x*) and *configuration* (***x***) for the collection of states of the set of automata of B, i.e. the collective network state. Starting from an initial configuration, ***x***^0^, the nodes of a BN are updated with a *synchronous* or *asynchronous* policy. The *dynamics* of B is thus defined by the temporal sequence of the 2^*n*^ possible configurations that ensue. The transitions between configurations can be represented as a *state transition graph* (STG), where each vertex is a configuration, and each directed edge denotes a transition from ***x***^*t*^ to ***x***^*t*+1^. The STG of B thus encodes the network’s entire *dynamical landscape*. Under the synchronous updating scheme (used in the studies reported in this paper) configurations that repeat, such that ***x***^*t*+*μ*^ = ***x***^*t*^, are known as *attractors*; *fixed point* when *μ* = 1; and *limit cycle*, with period *μ*, when *μ* > 1. The disconnected sub-graphs of a STG that lead to an attractor are known as *basins of attraction*. A BN B has a finite number of attractors, *b*, each denoted by $A_{i}\;:\;i=1,\ldots ,b$.

### 4.3. Effective connectivity

The *effective connectivity* (*k*_*e*_) tallies the expected number of inputs of an automaton *x*_*i*_ that are *minimally sufficient* to determine its state transitions. When a subset of such minimal inputs is in a certain state combination, the remaining inputs are effectively redundant—they can be in any state with no effect on the transition of *x*_*i*_. These effective inputs, or *enputs* for short, can be identified using the schema redescription methodology introduced by Marques-Pita & Rocha [25], which we illustrate next. The formula for the logic rule OR with two inputs can be written as $x=i_{1}\vee i_{2}$. The truth table for this expression can be redescribed as *wildcard schemata* as follows: *F*′_1_ = {(1, #), (#, 1)} and *F*′_0_ = {(0, 0)}, where *F*′_1_ denotes the set of wildcard schemata that prescribe transitions to 1 (ON), and, conversely, *F*′_0_ denotes the wildcard schemata prescribing transitions to 0 (OFF), a set that contains only one schema in this case. The wildcard symbol ‘#’ in a schema denotes a redundant input state. For example, (1, #) is interpreted as follows: given *i*_1_ = 1, then the transition *x*^*t*+1^ = 1 is guaranteed, regardless of the state of *i*_2_. A closer look at *F*′_1_ reveals that only one input is necessary to settle transitions to 1 (ON) in this example, and this is the case for the OR rule with any number of inputs. The entire set of schemata for a given automaton can be used to determine its effective connectivity. This requires the computation of the average *minimal* number of enputs necessary to determine its state transition. Effective connectivity is computed from the upper bound on *input redundancy* [25], yielding a sum of the minimal number of enputs required to settle each of the possible 2^*k*^ state transitions specified in the automaton’s LUT. This value is then divided by 2^*k*^ to obtain *k*_*e*_. For this computation, we iterate over the entire LUT of the automaton; for each LUT entry we accumulate the number of enputs of the wildcard schema matched, with the largest number of wildcard symbols; once all LUT entries have been processed, the final accumulated sum is divided by the LUT size. In our example *k*_*e*_ = 1.25. This is the case since *three* of the *four* look-up entries in the LUT have *one* of the inputs in the *on* state, which is sufficient to settle the transition, while one of the entries requires *two* (*i*_1_ = 0, *i*_2_ = 0), so in this case *k*_*e*_ = [(3 × 1) + (1 × 2)]/4; see [25] for details. Note that *k*_*e*_ ≤ *k* and that the higher the difference between *k*_*e*_ and *k*, the more canalization there is in the automaton rule, and, also, the lower the effective connectivity the automaton will have as a node in a BN.

Other measures of canalization in Boolean automata exist and have been linked to criticality, such as *average sensitivity* [57] and the more general *c-sensitivity* [71]. Effective connectivity presents several advantages over these measures. First and foremost, it is designed to capture collective canalization [25], a very common nonlinear phenomenon in automata whereby a subset of inputs *jointly* determine the state of an automaton, while rendering redundant the complement subset of inputs [6]. By contrast, sensitivity independently aggregates the influence (*activity*) of each individual input to an automaton. It is thus a linear measure of canalization. This means that effective connectivity provides a more nuanced and realistic measurement of canalization that includes nonlinear effects [24,70]. For instance, even for automata of *k* = 2, sensitivity does not discriminate between such common Boolean functions as conjunction/disjunction and proposition/negation: $s(x_{1}\wedge x_{2})=s(x_{1}\vee x_{2})=s(x_{1})=s(\neg x_{1})=1$. Effective connectivity, on the other hand, correctly accounts for the additional collective canalization that is present in the conjunction/disjunction (and other) functions: $k_{e}(x_{1}\wedge x_{2})=k_{e}(x_{1}\vee x_{2})=5/4=1.25$, while $k_{e}(x_{1})=k_{e}(x_{1})=1^{k}$. Since nonlinear, collective canalization increases with *k* [6,24], the finer characterization of the phenomenon provided by effective connectivity becomes more relevant as well. Interestingly, both sensitivity and effective connectivity can be easily computed from our schema description methodology [24], which is available in the CANA Python package [63]. Finally, ‘*c*-sensitivity’ [71] extends sensitivity to subsets of *c* inputs, but it results in a vector of *k* values for each *c*, which is much less amenable to the regression analysis of criticality boundaries we pursue in this study than is the scalar value measured by *k*_*e*_.

### 4.4. Generation of RBN ensembles

Each of the ensembles of RBNs that we produced for this study is characterized by a set of tuning parameters, namely (*k*, *k*_*e*_, *p*). The network connectivity *k* is a fixed (homogeneous) variable. This means that in our ensembles every node *x*_*i*_ is connected to *k* nodes. The effective connectivity is the mean value in a small interval (bin), and the bias is also fixed (homogeneous). Note that the values of these parameters are always homogeneously distributed, in alignment with the assumptions made by the ST in equation (1.1). For a given value combination of (*k*, *k*_*e*_, *p*), a single random BN is generated by choosing: (i) for each constituent node, a random set of *k* input nodes; and (ii) a random Boolean automaton with *k* inputs, output-bias *p* and effective connectivity in a small range *k*_*e*_ ± *ε* from an existing catalogue. The reason for binning *k*_*e*_ is that the possible values for this parameter vary significantly for each combination of *k* and *p*, which leads to a sparse matrix of viable ensembles (*k*, *k*_*e*_, *p*), where viability is determined by the existence of Boolean state transition rules that satisfy specific combinations of the parameter values (see electronic supplementary material, appendix S3 for further details). Thus, without loss of information, we bin *k*_*e*_ using a small bin size *ε* = 0.25, leading to *k*_*e*_ being homogeneously distributed in regular intervals of size Δ*k*_*e*_ = 0.5 and to a more dense matrix of viable ensembles. Because the values of *k*_*e*_ are binned, we refer to the *k*_*e*_ tuning parameter as 〈*k*_*e*_〉. Producing a random Boolean automaton with a given (*k*, *p*) is simple: (i) generate an all-zeros vector of length 2^*k*^; (ii) assign the state 1 (*on*) to (2^*k*^)*p* LUT random entries in the resulting vector; and (iii) assume the updated vector represents the state transitions of the automaton in the lexicographic order of input combinations. To control for *k*_*e*_, we generate a catalogue of Boolean automata with a large number of (*k*, *k*_*e*_, *p*) value combinations, from which automata with the appropriate parameter values are picked during the generation of the RBN ensembles. The catalogues for Boolean rules of *k* = 2, 3, 4 are exhaustive. For larger *k*, automata are first obtained by random generation for a given *k* and *p*, with their *k*_*e*_ subsequently computed. The number of possible automata for a given *k* and *p* is $\left(\begin{array}{c} 2^{k}\\ \;p(2^{k}) \end{array}\right)$. Thus, for *k* > 4, the catalogues contain a random sample of 10^4^ Boolean rules for each (*k*, *p*) if the total number possible is greater than 10^4^, and all the Boolean state transition rules otherwise. Additionally, to obtain automata with *k*_*e*_ in ranges essentially inaccessible to random generation via *k* and *p* alone, we use a genetic algorithm. We refer the interested reader to electronic supplementary material, appendix S3 for details. We have considered the following ranges for our tuning parameters: the number of nodes per network *N* = 100, *k* ∈ {2, 3, 4, 6, 8}, *p* = [0.01, 0.5] with Δ*p* = 1/2^*k*^, and 〈*k*_*e*_〉 = [1, *k*] with Δ*k*_*e*_ = 0.5. By sweeping the space of values for our ensemble parameters, we have generated a total of 266.4K RBNs.

### 4.5. Computation of the Derrida parameter

For a given BN, we compute the *ζ* parameter [18,49,50] by first generating *I* = 250 random initial configurations, producing an almost identical copy for each, where the copy differs only in the state of a small number *m* of states that have been perturbed (flipped). We set this value to be a random integer *m* ∈ [1, …, *N*/10]. Second, allowing the BN to advance each pair of initial configurations (original and perturbed) for *t* time steps, we set *t* = 1. Third, computing the Hamming distance between the two resulting configurations. Fourth, for each value of *m*, averaging the Hamming distances obtained in the previous step and plotting them against *m* to produce the Derrida plot. Finally, fifth, calculating *ζ* as the slope of the Derrida plot at the origin. A value of *ζ* = 1 indicates criticality. A value above (below) this is interpreted as meaning the BN is in the chaotic (stable) dynamical regime.

The Derrida parameter is a network measure that does not distinguish different roles nodes may play in a BN model, e.g. being an input node or a node regulated by other nodes. Thus, while input nodes receive only a self-loop, they are as likely to be perturbed as any other node when computing *ζ*. This is a reasonable procedure because perturbations to any node—input or not—can in principle propagate downstream.

### 4.6. Constrained search for best decision boundaries classifying the dynamical regime

The dataset we produce contains individual RBNs, each characterized by the independent variables *k*, *p*, and *k*_*e*_, and with one dependent variable with value one (1) if *ζ* > 1 (chaos), and zero (0) otherwise. We perform binary logistic regression to identify the decision boundary separating dynamic regimes using a set of predefined model classes. The general form of all models in every class is: *R*=step(logistic(model)), where the output of the logistic function is the probability that the dependent variable has value 1 (chaotic regime). The output of the step function is the predicted binary value of the dependent variable given a threshold *τ* = 0.5. If the output of the step function for the BN variables in a given model is greater than *τ* then the classifier predicts that BN is in the chaotic regime, and critical/stable otherwise. Each model tested belongs to one of the following model classes, where *κ* is the in-degree *k* in the ST or the mean effective connectivity 〈*k*_*e*_〉 in the CT, listed in increasing order of model complexity. Model complexity is defined by the number of terms and the number of predictors in each term (in that order),

4.1 $$\begin{array}{rl} & 1.\;c_{1}\kappa ;\\ & 2.\;c_{1}\kappa p(1-p);\\ & 3.\;c_{1}\kappa +c_{2p}(1-p);\\ & 4.\;c_{1}\kappa +c_{2}\kappa p(1-p);\\ & 5.\;c_{1}\kappa p(1-p)+c_{2p}(1-p);\\ & 6.\;c_{1}\kappa +c_{2}\kappa p(1-p)+c_{3p}(1-p). \end{array}$$

In our binary logistic regression, we use the *p*(1 − *p*) as a single independent variable accounting for the bias, rather than just *p* due to the principle of duality in Boolean logic. The coefficients derived for each *criticality model* are used to construct a decision surface. For this, the resulting equations have been manipulated so that the independent variables and their coefficients are on the left-hand side and the value (1) on the right-hand side, thus facilitating comparisons with the ST.

### 4.7. Performance measures

Mc-Fadden’s *R*^2^ is a standard goodness-of-fit measure used for logistic regression models. It is computed as one minus the ratio of the log-likelihood of the model to that of the intercept-only model [72]. The maximum value of this pseudo-*R*^2^ is 1. The MCC is ideal for computing classification performance in unbalanced scenarios [59], such as the one studied here, whereby there are many more instances of chaotic automata networks in the random ensembles than instances of stable network dynamics. Computed for the classifier using model predictions and test data, it is defined as a function of the number of true positives (TP), false positives (FP), true negatives (TN) and false negatives (FN): MCC = $(\mathrm{TP}\times \mathrm{TN}-\mathrm{FP}\times FN)/\sqrt{\left(\mathrm{TP}+\mathrm{FP})(\mathrm{TP}+\mathrm{FN})(\mathrm{TN}+\mathrm{FP})(\mathrm{TN}+\mathrm{FN}\right)}$ [59]. The MCC ranges between −1 and 1, where −1 indicates perfect opposite classification, 1 indicates perfect classification and 0 indicates random classification. Here, the positive label is associated with the chaotic dynamical regime *R* = 1, and the negative label with the stable (stable/critical) regime *R* = 0. The AUC is defined as a function of the true positive rate (TPR), the proportion of true positives in the total number of positive instances, false positive rate (FPR) and the proportion of false positives in the total number of negative instances, as follows: $\mathrm{AUC}=\int_{1}^{0}\mathrm{TPR}(T){\mathrm{FPR}}^{\prime }(T)\;dT$. The AUC ranges between 0 and 1, for perfectly incorrect and correct ranking of true class labels, respectively. A random classifier yields a value of 0.5. It is interpreted as the probability with which the classifier ranks positive instances (label 1) higher than negative instances (label 0) [73].

### 4.8. Nested fourfold cross-validation

The full dataset was randomly split into four non-overlapping equally sized partitions (75–25% training and testing splits). This was repeated four times, thus yielding *outer foldings*. A similar procedure was followed on each of the training splits, yielding a total of 16 training–testing pairs (see electronic supplementary material, appendix S1 for further details). Measures of classification and regression performance (as with the full dataset) on the testing splits were collected. The 16 sets of performance scores were averaged to produce an estimate of generalization performance score for each measure. Between-model comparisons were made using pair-sample *t*-tests because the two models were evaluated on the same set of 16 test folds. The paired *t*-tests were one-sided with the alternative hypothesis that the mean score of model 2 (〈*k*_*e*_〉) is greater than that of model 2 (*k*).

### 4.9. Symbolic regression

A supplementary study was performed using a different curve-fitting method to find the critical decision surface. We used symbolic regression (a type of unconstrained search), which is, in essence, a genetic programming algorithm [60]. The symmetric effect of the biases *p* and 1 − *p* on the Derrida parameter was used to prune the search space by considering 0 < *p* ≤ 0.5 only. Note that symbolic regression works in a much larger space of many function classes than the space of six model classes considered in our main methodology. Because of this, it can be hard to find an optimal function that is both consistent and guarantees minimal complexity. Furthermore, the obtained classifiers and coefficients can be hard to interpret in some cases. One of the relevant uses of this kind of method is to find different models for a given classification problem, for example, and compare them. One of the benefits of this is to help in determining suitable function classes to describe a classification decision boundary.

Symbolic regression was performed on our dataset from different (random) seeds eight times. We allowed for any formula in evolving populations that included basic arithmetic operators, coefficients, exponents and the sine, cosine and logarithmic functions. In every execution of the algorithm, we consistently obtained a classifier with the same function form based on an interaction between *k*_*e*_ and *p* with a coefficient that varied slightly in different runs. The ensembles were defined in the same way as in the main methods with the only difference that we used networks of size *N* = 48 instead of *N* = 100. The best classifier found was the function 3.125〈*k*_*e*_〉*p* = 1, with performance values very close to those of the CT. See electronic supplementary material, appendix S2 for further details.

## Endnotes

1 Automata models of biochemical regulation may include variables that can take multiple states (beyond the two in Boolean logic); however, there is always a larger BN that is isomorphic to any multi-state automata network [7,74].

2 Various sources of stochasticity can also be added to the updating scheme [5,25].

3 The theory was also expanded to allow *k* to be randomly sampled from scale-free distributions [28]. However, we do not consider such extreme heterogeneous connectivity in the present work because the experimentally validated systems biology networks we test are far from scale-free, with values of *k* that span little more than a scale. Moreover, our effective connectivity measure has an even smaller range (§2). See also §3 for of heterogeneous connectivity and possible expansions of the work.

4 Indeed, the specific positive coefficient *β* obtained (*β* = 0.09 in our analysis) is arbitrary and selected in the optimization search from infinite positive values leading to the same performance; all that matters for this model is that the critical value *k*^*c*^ ≤ 0, so that every network (*k* > 0) is predicted to be chaotic.

5 Notice that a slightly different critical boundary defines the ST in equation (1.1) (*c*_1_ = 2) than what we obtained in equation (2.1) by optimizing model class (2) for *κ* ≡ *k* against random ensemble data (*c*_1_ = 1.49). This is likely because the ST was derived theoretically, while our model 2 is optimized for empirical data circumscribed to a finite *k* range. Indeed, the theoretical ST is not optimal in this range, and leads to slightly worse classification performance than the optimized version of equation (2.1) on the RBN ensembles: MCC = 0.73 and *R*^2^ = 0.28.

6 Since the theoretically derived ST (equation (1.1)) underperforms its empirical counterpart, there is no need to report significance for the former.

6 $\tilde{k_{e}^{C}}=Q_{1}=1.25$ (median and lower quartile) in the distribution of *k*_*e*_ for Cell Collective automata because almost half of the automata in data are logical AND or OR functions of *k* = 2 (see figure 6*a*) that have exactly *k*_*e*_ = 1.25 (see figure 6*b*). In addition, automata with *k* > 2 frequently have *k*_*e*_ = 1.25, e.g. when all inputs are fully redundant except for two that implement a logical AND or OR.

8 The distribution of *k*_*e*_ is right-skewed and leptokurtic with skewness and kurtosis values that suggest a log-normal distribution. But such a heterogeneous distribution is still circumscribed to a very small dispersion range, as seen by the narrow $\mathrm{IQR}(k_{e}^{C})$ and boxplots in figure 6.

9 We pursue both class-constrained regression (equations (4.1)) and unconstrained symbolic regression analyses that lead to almost identical critical boundaries in the same model class (§3.2).

10 In the space of $2^{2^{k}}$ possible logical rules for a given *k*, there are only (2^*k*^) − 2 distinct values of *p* when tautologies and contradictions are ignored, and this number is halved when accounting for bias symmetry in Boolean functions. Thus, automata with very different logic can have the same *k* and *p*; *k*_*e*_, on the other hand, allows us to differentiate when such automata have distinct canalization logic as shown in Figure S3-1 in the electronic supplementary material.

## Data accessibility

We include an electronic supplementary material document with further theoretical, methodological and data details. In addition, we also include the Boolean automata source catalogues, and the R scripts used to produce all of the reported results. See the electronic supplementary material for details.
